# Interleukin-6 Is a Potential Biomarker for Severe Pandemic H1N1 Influenza A Infection

**DOI:** 10.1371/journal.pone.0038214

**Published:** 2012-06-05

**Authors:** Stéphane G. Paquette, David Banner, Zhen Zhao, Yuan Fang, Stephen S. H. Huang, Alberto J. Leόn, Derek C. K. Ng, Raquel Almansa, Ignacio Martin-Loeches, Paula Ramirez, Lorenzo Socias, Ana Loza, Jesus Blanco, Paola Sansonetti, Jordi Rello, David Andaluz, Bianche Shum, Salvatore Rubino, Raul Ortiz de Lejarazu, Dat Tran, Giovanni Delogu, Giovanni Fadda, Sigmund Krajden, Barry B. Rubin, Jesús F. Bermejo-Martin, Alyson A. Kelvin, David J. Kelvin

**Affiliations:** 1 Division of Experimental Therapeutics, Toronto General Hospital Research Institute, University Health Network, Toronto, Ontario, Canada; 2 Institute of Medical Science, Faculty of Medicine, University of Toronto, Toronto, Ontario, Canada; 3 International Institute of Infection and Immunity, Shantou University Medical College, Shantou, Guangdong, China; 4 Department of Immunology, Faculty of Medicine, University of Toronto, Toronto, Ontario, Canada; 5 Infection and Immunity Medical Investigation Unit, Hospital Clínico Universitario - Instituto de Estudios de Ciencias de la Salud de Castilla y Leόn, Valladolid, Spain; 6 Critical Care Centre Parc Tauli, Sabadell University Hospital, Sabadell, Spain; 7 Critical Care Department, Hospital Universitario La Fe - Sociedad Española de Medicina Intensiva, Crìtica y Unidades Coronarias, Valencia, Spain; 8 Critical Care Department, Hospital Son Llatzer - Sociedad Española de Medicina Intensiva, Crìtica y Unidades Coronarias, Palma de Mallorca, Spain; 9 Critical Care Department, Hospital N Sra de Valme - Sociedad Española de Medicina Intensiva, Crìtica y Unidades Coronarias, Sevilla, Spain; 10 Critical Care Department, Hospital Universitario Rio Hortega - Salud de la Junta de Castilla y León - Sociedad Española de Medicina Intensiva, Crìtica y Unidades Coronarias and El Centro de Investigación Biomédica en Red de Enfermedades Respiratorias (Instituto de Salud Carlos III), Valladolid, Spain; 11 Instituto di Microbiologica, Università Cattolica del Sacro Cuore, Rome, Italy; 12 Critical Care Department, area General, Hospital Vall d’Hebron, Institut de Recerca Vall d’Hebron-Universitat Autònoma de Barcelona, El Centro de Investigación Biomédica en Red de Enfermedades Respiratorias - Sociedad Española de Medicina Intensiva, Crìtica y Unidades Coronarias, Barcelona, Spain; 13 Critical Care Department, Hospital Clínico Universitario- Salud de la Junta de Castilla y León/Sociedad Española de Medicina Intensiva, Crìtica y Unidades Coronarias, Valladolid, Spain; 14 Department of Microbiology, St. Joseph’s Health Centre, Toronto, Ontario, Canada; 15 Sezione di Microbiologia Sperimentale e Clinica, Dipartimento di Scienze Biomediche, Universita’ degli Studi di Sassari, Sassari, Italy; 16 Division of Infectious Diseases, Department of Pediatrics, The Hospital for Sick Children, University of Toronto, Toronto, Ontario, Canada; 17 Division of Vascular Surgery, Peter Munk Cardiac Centre, Toronto General Hospital Research Institute, University Health Network, Toronto, Ontario, Canada; 18 Immune Diagnostics & Research, Toronto, Ontario, Canada; German Primate Center, Germany

## Abstract

Pandemic H1N1 influenza A (H1N1pdm) is currently a dominant circulating influenza strain worldwide. Severe cases of H1N1pdm infection are characterized by prolonged activation of the immune response, yet the specific role of inflammatory mediators in disease is poorly understood. The inflammatory cytokine IL-6 has been implicated in both seasonal and severe pandemic H1N1 influenza A (H1N1pdm) infection. Here, we investigated the role of IL-6 in severe H1N1pdm infection. We found IL-6 to be an important feature of the host response in both humans and mice infected with H1N1pdm. Elevated levels of IL-6 were associated with severe disease in patients hospitalized with H1N1pdm infection. Notably, serum IL-6 levels associated strongly with the requirement of critical care admission and were predictive of fatal outcome. In C57BL/6J, BALB/cJ, and B6129SF2/J mice, infection with A/Mexico/4108/2009 (H1N1pdm) consistently triggered severe disease and increased IL-6 levels in both lung and serum. Furthermore, in our lethal C57BL/6J mouse model of H1N1pdm infection, global gene expression analysis indicated a pronounced IL-6 associated inflammatory response. Subsequently, we examined disease and outcome in IL-6 deficient mice infected with H1N1pdm. No significant differences in survival, weight loss, viral load, or pathology were observed between IL-6 deficient and wild-type mice following infection. Taken together, our findings suggest IL-6 may be a potential disease severity biomarker, but may not be a suitable therapeutic target in cases of severe H1N1pdm infection due to our mouse data.

## Introduction

Influenza is an acute viral infection of the respiratory tract caused by influenza type A, B, and C viruses of the *Orthomyxoviridae* family [1]. Influenza is typically spread through seasonal or sporadic epidemics, but the periodic emergence of novel influenza strains can result in widespread pandemics capable of extensive morbidity and mortality. In early 2009 pandemic H1N1 influenza A (H1N1pdm), a novel influenza A H1N1 strain of swine-origin, emerged from Mexico and rapidly spread worldwide [2]. Although the pandemic period of H1N1pdm has now passed [3], H1N1pdm remains a dominant circulating influenza strain in various parts of the world.

Infection with H1N1pdm has resulted in diverse clinical outcomes. The vast majority of reported cases have been mild and self-limiting [4]–[6]; typical symptoms have included fever, sore throat, malaise, and headache [6], [7]. However, a subset of cases have been characterized by serious illness, often requiring hospitalization and mechanical ventilator support [2], [6], [8], [9]. Common complications in such severe cases of H1N1pdm infection have included: severe hypoxemia, shock, pneumonia, and acute respiratory distress syndrome (ARDS) [2], [8], [9]. Nonpulmonary acute organ dysfunction has also been reported [9], [10]. Importantly, the triggers for severe illness are not completely understood and the host immune response to H1N1pdm infection remains to be comprehensively characterized.

Inflammation is a rapid and non-specific host defense mechanism against infection that is tightly regulated by a network of inflammatory mediators, which includes cytokines such as interleukin-6 (IL-6) [11]–[13]. IL-6 is a pleiotropic cytokine significantly implicated in various facets of the immune response, including a prominent role in inflammation. IL-6 is the chief mediator of the acute phase response and fever [14]. IL-6 also has important involvement in many pathogenic inflammatory states: IL-6 levels are elevated and correlate with sepsis severity and mortality [15] and IL-6 has been implicated in the cytokine storm following avian influenza A H5N1 and severe acute respiratory syndrome (SARS) infection [16]–[18]. In contrast, studies have also shown that IL-6 may play roles in regulating and limiting inflammation, with mice deficient for IL-6 displaying more pronounced inflammation and neutrophilia in response to aerosolized endotoxin [19]. Within the context of the lung, IL-6 promotes pulmonary inflammation and IL-6 levels correlate with severity and mortality of ARDS [20], [21].

IL-6 levels have been associated with symptom duration and severity in cases of seasonal influenza infection [22]–[24]. Recently, increased IL-6 expression was observed in severe cases of H1N1pdm infection, with a significant inverse association between IL-6 and arterial oxygen levels in patients hospitalized with H1N1pdm infection [25]. Given the known involvement of IL-6 in pathogenic lung inflammation, we sought to investigate the role of IL-6 in severe H1N1pdm infection, and potential contributions to disease. Here, we report increased IL-6 expression in both humans hospitalized with H1N1pdm infection and in multiple mice strains infected with H1N1pdm (A/Mexico/4108/2009 (H1N1pdm)), which implicated IL-6 in the host response to H1N1pdm infection. We then explored the effect of IL-6 deficiency on disease in H1N1pdm infected IL-6−/− mice. Our results indicated that IL-6 expression was strongly associated with disease severity although loss of IL-6 did not alter disease outcome in mice. This suggested IL-6 as a marker of severe influenza H1N1pdm disease but not a potential therapeutic target which has important implications on the design and administration of influenza A therapeutics.

## Results

### Serum IL-6 Levels Associate Strongly with Disease Severity in Patients Hospitalized with H1N1pdm Infection

**Figure 1:**
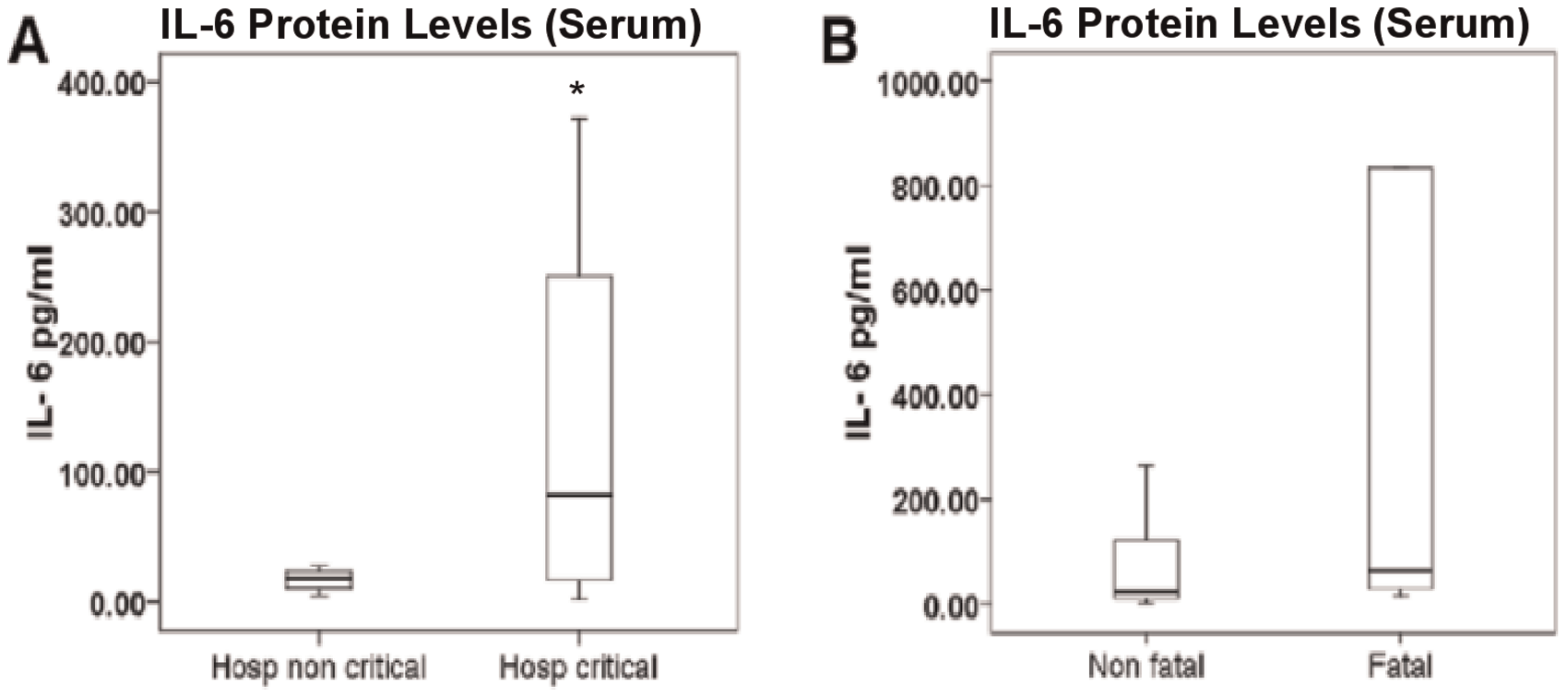
IL-6 levels associated strongly with disease severity in patients hospitalized with H1N1pdm infection. Serum IL-6 levels in hospitalized patients with laboratory-confirmed H1N1pdm infection. Comparison between different groups: between patients requiring critical (n = 35) or non-critical (n = 10) care (A) and between patients who survived (n = 35) or died (n = 10) (B). Mann-Whitney U test was applied to assess statistical significance of differences between groups; p-values <0.05 are indicated by an asterisk (*).

Increased IL-6 expression has been reported following seasonal influenza infection, as well as in severe cases of H1N1pdm infection [22]–[25]. We investigated IL-6 expression in humans hospitalized with H1N1pdm infection and further analyzed patient outcome with clinical signs. A total of 45 hospitalized patients with laboratory-confirmed H1N1pdm infection were included in our analysis. Serum samples were collected within 48 hours of hospital admission and IL-6 protein concentrations were measured. IL-6 concentrations were found to be significantly higher in patients who required critical care support (n = 35) compared to patients who did not (n = 10) (Figure 1A). Also, IL-6 concentrations were higher in patients who died (n = 10) compared to survivors (n = 35), although differences were not statistically significant (p = 0.065) (Figure 1B). Binary logistic regression (BLR) analysis demonstrated that levels of IL-6 (log_10_ IL-6 (pg/mL)) adjusted by age and sex were significantly associated with increased risk of fatal outcome [Odds Ratio (95% Confidence Interval); p-value]: [2.63 (1.06–6.49); 0.036]. Taken together, these results revealed a strong association of IL-6 patient concentration to disease severity in people hospitalized with H1N1pdm infection.

### Severe H1N1pdm Infection Triggers Increased IL-6 Expression in mice

Our human clinical data implicated IL-6 in H1N1pdm infection. We next investigated IL-6 expression in mouse models of severe H1N1pdm infection. To observe possible strain variation [26], we infected three mouse strains, C57BL/6J mice (n = 17), BALB/cJ mice (n = 17), and B6129SF2/J mice (n = 17), intransally with 10^4^ EID_50_ A/Mexico/4108/2009 (H1N1pdm), and observed survival, weight loss, and IL-6 expression for fourteen days pi.

**Figure 2:**
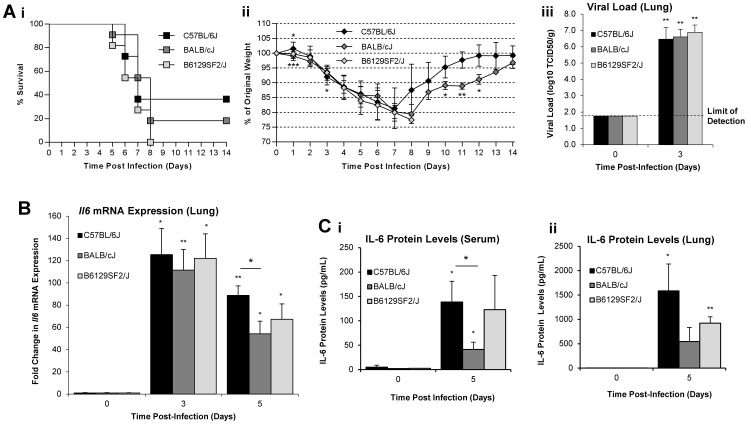
Severe A/Mexico/4108/2009 (H1N1pdm) infection triggered increased IL-6 expression in C57BL/6J, BALB/cJ, and B6129SF2/J mice. Survival curves for C57BL/6J (closed squares), BALB/cJ (grey squares), and B6129SF2/J (open squares) mice infected intranasally with 10^4^ EID_50_ A/Mexico/4108/2009 (H1N1pdm). n = 11 for all three groups. The logrank test (α = 0.05) was used to ascertain significance in differences in survival (A_i_) [43]. Average weight curve for C57BL/6J (closed diamonds), BALB/cJ (grey diamonds), and B6129SF2/J (open diamonds) mice. Vertical error bars indicate ±1 standard deviation. n = 17 for all groups. Asterisks below the curves indicate significant difference between C57BL/6J weights and BALB/cJ weights, asterisks above the curves indicate significant difference between C57BL/6J weights and B6129SF2/J weights. No significant differences between BALB/cJ and B6129SF2/J weights (A_ii_). Viral load in lung homogenates collected at days 0 and 3 pi. n = 3 for all groups. Infection of Madin-Darby Canine Kidney cells was employed to measure viral titers. The assay had a limit of detection of 10^1.75^ TCID_50_/g of lung tissue, indicated by the dashed line. Vertical error bars indicate +1 standard deviation (A_iii_). *Il6* mRNA expression profiling in lung homogenates by qRT-PCR. Results are expressed as fold changes over expression in uninfected day 0 pi controls. n = 3 for each group. Vertical error bars indicate +1 standard deviation. (B). IL-6 expression levels in serum and lung homogenates. n = 3 for each group. Vertical error bars indicate +1 standard deviation (C_i&ii_). The two-tailed, two-sample unequal variances Student’s t-test was used to ascertain significance (p-value <0.05 = *, p-value <0.01 = **, p-value <0.001 = ***).

H1N1pdm infection caused severe disease in mice of all three groups (Figure 2A). Dramatic declines in weight were observed as of day 3 post-infection (pi) through to days 7–8 pi, with reductions in percent survival as of day 5 pi. Final percent survival was 36.4% (4/11), 18.2% (2/11), and 0% (0/11) for C57BL/6J, BALB/cJ, and B6129SF2/J mice, respectively. Interestingly, weight loss in BALB/cJ mice slowed slightly at days 5–6 pi, delaying drops in percent survival by one day relative to C57BL/6J and B6129SF2/J mice. Yet, no significant differences in survival curves were observed (α = 0.05). Similar viral loads were detected in the lungs of infected mice at day 3 pi.

We next evaluated *Il6* gene regulation at the mRNA level following H1N1pdm infection. Total RNA was extracted from lung homogenates and assayed by quantitative real-time RT-PCR (qRT-PCR) for fold changes in *Il6* mRNA expression at days 0, 3, and 5 pi (n = 3). At both day 3 and 5 pi, *Il6* mRNA was significantly increased in the lungs of all mouse strains following H1N1pdm infection (Figure 2B) peaking on day 3 pi. No significant differences were seen among the mouse groups on day 3, although significant differences were seen day 5 pi between C57BL/6J and BALB/cJ mice.

To measure IL-6 cytokine protein levels, lung homogenates and serum were collected at days 0 and 5 pi, n = 3 at each time-point. Dramatic increases in IL-6 cytokine levels were observed at day 5 pi (Figure 2C). Among the three groups, BALB/cJ mice were the weakest producers of IL-6 in both serum and lung. Serum IL-6 protein levels were significantly higher in C57BL/6J mice than in BALB/cJ mice. Similarly, C57BL/6J mice had higher lung IL-6 protein levels than BALB/cJ mice, although differences were not statistically significant (p = 0.063). No significant differences in IL-6 protein levels were observed in B6129SF2/J mice in relation to C57BL/6J or BALB/cJ mice, with p-values >0.10 in all cases. Levels of proinflammatory cytokines tumor necrosis factor alpha (TNF-α) and monocyte chemotactic protein-1 (MCP-1) were also increased following infection, with no significant differences between groups (data not shown). Taken together, our results revealed similar disease severity in C57BL/6J, BALB/cJ, and B6129SF2/J H1N1pdm infected mice. Furthermore, increased IL-6 expression was consistently observed in all groups, suggesting an important role in host responses across mice of different genetic backgrounds.

### C57BL/6J mice Elicit IL-6 Associated Inflammatory Response to H1N1pdm Infection

We observed increased IL-6 levels in response to severe H1N1pdm infection in mice. This led us to investigate by functional genomics the impact of IL-6 on the inflammatory response during H1N1pdm infection. C57BL/6J mice were infected intranasally with a lethal dose (10^5^ EID_50_) of A/Mexico/4108/2009 (H1N1pdm) (Figure S1). Lung homogenates from infected animals were collected daily for RNA extraction and subsequent analysis of gene expression from days 0 to 6 pi, n = 3 at each time point.

**Figure 3:**
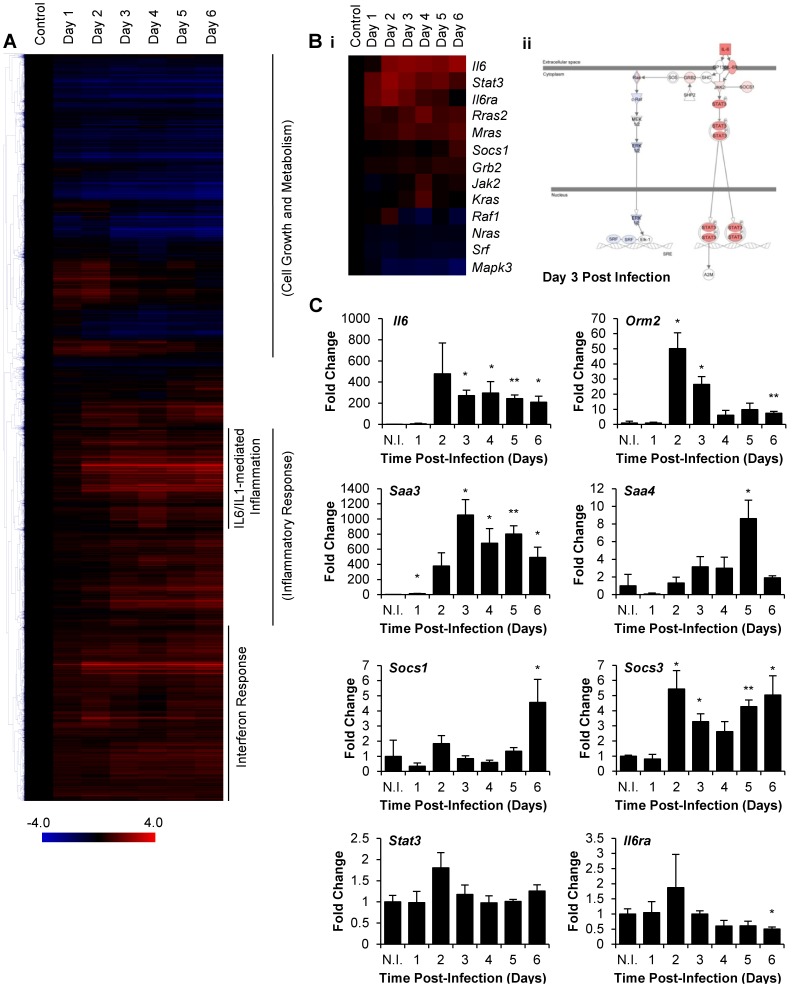
C57BL/6J mice elicited IL-6 associated inflammatory response to A/Mexico/4108/2009 (H1N1pdm) infection. Global gene expression profiling in lungs of C57BL/6J mice infected intranasally with 10^5^ EID_50_ A/Mexico/4108/2009 (H1N1pdm). Probes for significantly differentially expressed genes were subjected to one-way hierarchical clustering analysis (Pearson’s correlation). The most significant gene networks (Inflammatory Response, Cell Growth and Metabolism) or most prominent represented canonical pathways (IL-1 and IL-6-mediated Inflammation, Interferon Response), as determined by IPA, are indicated for each cluster (A). Expression data of genes associated with IL-6 signalling, as determined by IPA, profiled over the course of infection (B_i_). Visual representation of IL-6 signalling pathway, as represented by IPA, at day 3 pi. Overlaid colors represent gene regulation status; upregulated genes are red, downregulated genes are blue. Color intensity correlates with the magnitude of change in gene expression (B_ii_). Gene expression data validation by qRT-PCR for *Il6*, IL-6 signalling genes *Stat3* and *Il6ra*, and IL-6 response genes *Orm2*, *Saa3*, *Saa4*, *Socs1*, and *Socs3*. Results are expressed as fold changes over expression in non-infected controls. Reported values for each time point are the average of three samples with +1 standard deviation indicated by vertical error bars. N.I. indicates non-infected controls. The two-tailed, two-sample unequal variances Student’s t-test was used to ascertain significance (p-value <0.05 = *, p-value <0.01 = **) (C).

Statistical analysis of gene expression data by Extraction of Differential Gene Expression (EDGE) software identified 7,803 probes for significantly differentially regulated genes. Hierarchical clustering by Pearson’s correlation revealed several groups of co-ordinately expressed genes. Functional classification with Ingenuity Pathway Analysis v9.0 (IPA) delineated three prominent functional clusters: cell growth and metabolism, interferon response, and inflammatory response (Figure 3A). The cell growth and metabolism cluster was characterized by a gradual decline in gene expression over the course of infection. The interferon and inflammatory response clusters were both rapidly upregulated following infection: by day 2 pi, expression of the majority of genes in both clusters had measurably increased. However, while expression levels of interferon response genes had increased early and subsequently stabilized by day 3 pi, inflammatory response gene expression levels increased more gradually and stabilized later than day 3 pi. Within the inflammatory response cluster was a group of genes distinguishable for markedly increased expression. Functional analysis with IPA revealed genes associated with multiple inflammatory pathways regulated by interleukin-1β (IL-1β) and IL-6. Global microarray expression analysis displayed hallmarks of the early host response to viral infection and highlighted the prominent and rapid IL-1β/IL-6 mediated response.

To evaluate the status of IL-6 signalling during infection, associated genes as determined by IPA were investigated (Figure 3Bi). Early in infection, downstream signalling machinery, most notably IL-6 binding receptor subunit alpha (*Il6ra*) and intracellular signal transducer and activator of transcription 3 (*Stat3*), were upregulated, with peak upregulation observed at day 2 pi. This preceded upregulation of *Il6*, of which expression plateaued only by day 3 pi. IL-6 inducible negative regulator of Janus kinase 2 (*Jak2*), suppressor of cytokine signalling 1 (*Socs1*), was upregulated late following infection, at day 6 pi. Meanwhile, mitogen-activated protein kinase 3 (*Mapk3*) and serum response factor (*Srf*) were persistently downregulated throughout infection. Visual representation of the IL-6 signalling pathway reported by IPA at day 3 pi revealed upregulation of the JAK-STAT3 axis of IL-6 signalling, while the extracellular-signal-regulated-kinase (ERK)-mediated axis was downregulated (Figure 3Bii). Complementary analysis of *Il6*, *Stat3*, and *Il6ra* gene expression by qRT-PCR similarly identified peak upregulation of *Stat3* and *Il6ra* early at day 2 pi, and persistent upregulation of *Il6* throughout infection (Figure 3C). Genes associated with JAK-STAT3-mediated IL-6 signalling were upregulated early and throughout infection.

IL-6 has diverse functions, among which is positive regulation of the acute phase response [14]. To verify functional signalling by IL-6, the expression status of genes associated with the acute phase response was assessed. Using DAVID Bioinformatics Resource v6.7 [27], [28], multiple functional classifications, most notably Gene Ontology Biological Process, Panther, and Kyoto Encyclopaedia of Genes and Genomes were used to identify functionally related gene networks from all 7,803 probes for significantly differentially regulated genes. Gene Ontology Biological Process classification GO:0002526– acute inflammatory response was selected as a suitable representation of acute phase response effector genes; the network was upregulated throughout infection (Table S1). Results were consistent amongst all classification systems (data not shown). Complementary analysis by qRT-PCR of known IL-6-inducible genes orosomucoid 2 (*Orm2*), serum amyloid A 3 (*Saa3*), serum amyloid A 4 (*Saa4*), *Socs1*, and suppressor of cytokine signalling 3 (*Socs3*) confirmed increased IL-6- inducible gene expression (Figure 3C).

Analysis of gene expression data in a lethal mouse model of H1N1pdm revealed a cluster of co-ordinately expressed genes associated with IL-1β/IL-6 mediated inflammation, identified upregulation of genes responsible for IL-6 JAK/STAT3-mediated signalling, and confirmed increased IL-6 signalling with observed increases in expression of known IL-6-regulated pathways. These results suggested a prominent role for IL-6 associated inflammation in the host response to H1N1pdm infection in C57BL/6J mice.

### IL-6 Deficiency does not Significantly Impact H1N1pdm Infection Outcome in Mice

Increased IL-6 expression in both patients hospitalized with H1N1pdm and mouse models of H1N1pdm implicated IL-6 in the host response to infection. Given the known proinflammatory properties of IL-6 [20], [21], we hypothesized that IL-6 may play a pathogenic role in H1N1pdm infection. Thus, abrogation of IL-6 would reduce symptom severity and improve clinical outcome. Wild-type C57BL/6J (IL-6 wild-type) mice (n = 22) and IL-6−/− mice (n = 34) were infected intranasally with 10^4^ EID_50_ A/Mexico/4108/2009 (H1N1pdm), and observed for fourteen days pi.

**Figure 4:**
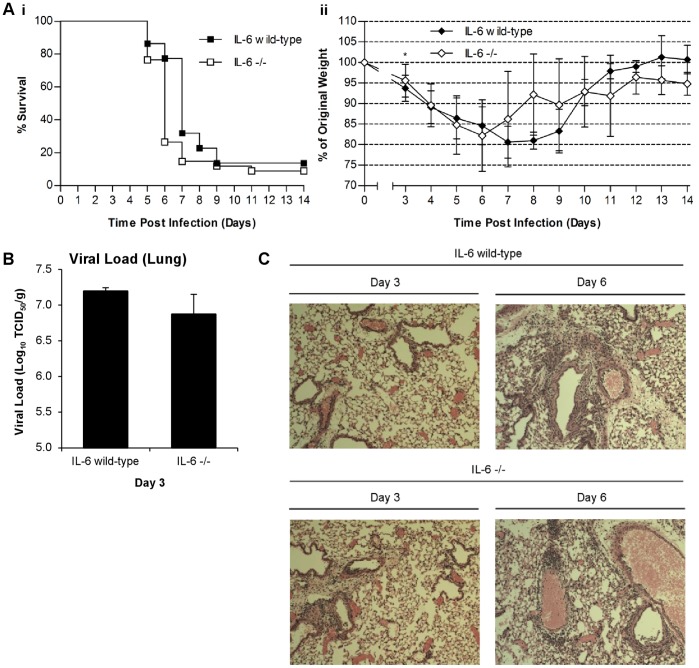
Loss of IL-6 expression in IL-6−/− mice did not significantly impact disease severity following A/Mexico/4108/2009 (H1N1pdm) infection. Survival curves for IL-6 wild-type (closed squares) and IL-6−/− (open squares) mice infected intranasally with 10^4^ EID_50_ A/Mexico/4108/2009 (H1N1pdm). n = 34 for IL-6−/− mice and n = 22 for IL-6 wild-type mice. The logrank test (α = 0.05) was used to ascertain significance in differences in survival (A_i_) [43]. Average weight curve for IL-6 wild-type (closed diamonds) and IL-6−/− (open diamonds) mice. Vertical error bars indicate ±1 standard deviation. The two-tailed, two-sample unequal variances Student’s t-test was used to ascertain significance (p-value <0.05 = *). n = 40 for IL-6−/− mice and n = 35 for IL-6 wild-type mice (A_ii_). Viral load in lung homogenates of IL-6−/− and IL-6 wild-type mice. Lung tissue collected at day 3 pi. n = 3 for IL-6−/− mice and n = 3 for IL-6 wild-type mice. Vertical error bars indicate +1 standard deviation. The two-tailed, two-sample unequal variances Student’s t-test was used to ascertain significance. Viral load expressed in log_10_ (B). Lung histology collected from IL-6 wild-type and IL-6−/−. Samples collected at both day 3 and day 6 pi (C).

IL-6 wild-type and IL-6−/− mice both exhibited severe illness following infection (Figure 4A). As of day 5 pi, weight loss in both groups resulted in marked decreases in survival. Percent survival levelled by day 9 pi at approximately 14% and 12% for IL-6 wild-type and IL-6−/− mice, respectively. Differences in survival and weight curves were not statistically significant (α = 0.05) between IL-6−/− and IL-6 wild-type mice. However, IL-6−/− mice tended to lose weight faster than IL-6 wild-type mice, causing IL-6−/− percent survival to drop one day prior to IL-6 wild-type percent survival. For both groups weight loss was observed until days 6–7 pi, followed by a recovery phase. Of the surviving mice, IL-6 wild-type (n = 3/22) and IL-6−/− mice (n = 3/34) recovered weights to near 100% and 95%, respectively. Taken together, these data showed no significant differences in the clinical outcome of H1N1pdm infected IL-6 wild-type and IL-6−/− mice.

To evaluate the effect IL-6 deficiency on viral load in the host, lung tissues were collected and homogenized day 3 pi, and viral loads were measured (Figure 4B). Average viral loads (n = 3) were 10^7.2^ and 10^6.9^ tissue culture infectious dose (TCID)_50_/g for IL-6 wild-type and IL-6−/− mice, respectively. Differences in viral loads were not statistically distinguishable (α = 0.05), although IL-6−/− mice did exhibit slightly lower viral loads.

To identify pathological differences resulting from IL-6 deficiency, lung tissues were collected at days 3 and 6 pi and examined (Figure 4C). At day 3 pi, both IL-6 wild-type and IL-6−/− exhibited alveolar thickening and minimal infiltration, with more infiltration observed in IL-6−/− mice. By day 6 pi, alveolar thickening was more prominent and infiltration more pronounced in both groups where the lung tissues were not markedly distinguishable. Taken together, the results from the IL-6−/− mice compared to IL-6 wild-type mice during H1N1pdm infection revealed no marked differences in survival, weight loss, or pathology.

## Discussion

Increased IL-6 expression and protracted pulmonary inflammation have been reported in critically ill H1N1pdm infected patients [6], [8], [9], [25]. We sought to investigate the role of inflammatory mediator IL-6 in severe H1N1pdm infection. Among humans hospitalized with H1N1pdm infection, we found serum IL-6 levels associated strongly with requirement of critical care admission and fatal outcome. Consistent with clinical observations, we found that IL-6 was increased during the host response to H1N1pdm infection in mice. However, infection of IL-6−/− mice resulted in disease indistinguishable from that in IL-6 wild-type mice, as measured by survival, weight loss, viral load, and pathology. Our findings suggest that IL-6 does not play an essential non-redundant role in the host response to H1N1pdm infection in mice. Furthermore, these results indicate more research is needed for the suitability of IL-6 as a therapeutic target and disease severity biomarker.

Severe H1N1pdm infection in humans is characterized by extensive pulmonary inflammation which can lead to ARDS and death [6], [8], [9]. Clinically, we found that IL-6 levels associated strongly with disease severity in hospitalized patients which was also mirrored in three mouse strains, suggesting a potential mechanism of disease. Cytokine storm is the induction of a protracted systemic inflammatory response stemming from cytokine dysregulation [29]. The resulting inflammation can cause extensive tissue damage, as has been observed during avian influenza A H5N1 and SARS infection [18], [30]–[32]. IL-6 has been implicated in both avian influenza A H5N1- and SARS-induced cytokine storms [16]–[18], [33], and from our data in humans we hypothesized that IL-6 would have a similar damaging role during severe H1N1pdm infection.

The genetic background of humans, mice and other animals are a significant factor leading to the type of immune response mounted toward pathogens [34]–[36]. To determine if IL-6 expression following H1N1pdm infection was dependent on the genetic background of the host, we investigated IL-6 levels in three mouse strains infected with H1N1pdm: C57BL/6J, BALB/cJ and B6129SF2/J. Previously, Otte et al. reported that BALB/cJ mice were less susceptible than C57BL/6J mice to H1N1pdm infection and speculated that this may be due to dampened cytokine expression in C57BL/6J mice [26]. In contrast, we found that C57BL/6J, BALB/cJ, and B6129SF2/J mice were similarly susceptible to A/Mexico/4108/2009 (H1N1pdm) infection. No statistically significant differences in disease severity were observed based on survival, weight loss, or lung viral load, although B6129SF2/J mouse final percent survival (0%) was markedly lower than that observed in C57BL/6J (36.4%) and BALB/cJ (18.2%) mice. Additional experiments, such as LD_50_ studies, may reveal significant differences in survival between mouse strains and clarify the potential genetic determinants of disease severity. Marked upregulation of IL-6 expression, both at the RNA and protein levels, was observed in mice of all three genetic backgrounds. Interestingly, we found C57BL/6J mice to be the strongest IL-6 producers while BALB/cJ mice were the weakest, suggesting cytokine responses may not have been dampened in C57BL/6J mice. Serum IL-6 protein levels were significantly higher in C57BL/6J mice than in BALB/cJ mice. No other differences were statistically significant but larger studies may further resolve differences in IL-6 responses between mouse strains. In summary, we found from our strain comparison studies that all three mouse strains had similar virus susceptibility, disease severity as well as induced levels of IL-6. Our findings implicated IL-6 in the host response to H1N1pdm in mice and also suggested C57BL/6J mice to be a suitable genetic background for IL-6 studies.

Global gene expression studies by microarray further underlined the importance of IL-6 in C57BL/6J mice as IL-6-mediated inflammatory response genes were prominently upregulated. Our microarray experiments were performed at a higher infection dose than our strain comparison studies (10^5^ EID_50_, compared to 10^4^ EID_50_). Since differences in infection dose could alter gene expression profiles in infected mice, we also investigated gene expression by real-time PCR for mice infected with 10^4^ EID_50_. At both 10^4^ EID_50_ and 10^5^ EID_50_, we consistently observed significant upregulation of *Il6* mRNA. Furthermore, we found IL-6 protein levels to be elevated in mice infected with 10^4^ EID_50_. Taken together, these results suggested a consistent role for IL-6 in response to H1N1pdm infection at either 10^4^ or 10^5^ EID_50_.

To investigate the immune mechanisms of severe H1N1pdm infection we employed an IL-6−/− mouse model, but did not observe significant differences in survival or weight loss compared to wild-type. We also found no differences in viral load or lung pathology, suggesting IL-6 did not play a direct role in the viral clearance or in the pathological destruction associated with the inflammatory process, despite the known proinflammatory roles of IL-6 [14], [15]. Since mice are often used for preliminary testing of therapeutic candidates for human diseases, results of our mouse IL-6−/− model suggest targeting IL-6 may not have the intended therapeutic benefit.

While the IL-6−/− mouse model has repeatedly been used to identify unique functions of IL-6 in immune responses to infection [37]–[40], additional proinflammatory cytokines may drive inflammation in the absence of IL-6. Specifically, IL-1β and TNF-α signalling are negatively regulated by IL-6 [19], [41] and may compensate for the loss of IL-6, which could explain why IL-6−/− mice were not protected during H1N1pdm infection. The potentially offsetting contributions of IL-1β and TNF-α in the absence of IL-6 should be investigated further.

Although C57BL/6J mice were the strongest IL-6 producers in our strain comparison studies, Otte and colleagues concluded that C57BL/6J mice are low cytokine producers. With this conclusion in mind, it is possible that dampened cytokine responses in C57BL/6J mice could minimize the role of hypercytokinemia during H1N1pdm infection. IL-6 deficiency in C57BL/6J background may not have affected survival and/or disease severity where it would have in another mouse background. Furthermore, C57BL/6J mice are predisposed to Th1-type immune responses whereas BALB/cJ mice are predisposed to Th-2 type immune responses. Although IL-6 deficiency had no effect in Th1-type skewed C57BL/6J mice, the role of IL-6 could be significantly different in Th2-type skewed BALB/cJ mice. It is important that future studies investigate IL-6 deficiency in other mouse strains to determine what impact host genetic background has on the protective or pathogenic roles of IL-6.

In humans, we found that serum IL-6 levels were significantly higher in critically ill hospitalized patients compared to non-critical patients. Among critically ill patients, we also observed a trend of increased IL-6 in nonsurvivors compared to survivors. Similarly, we detected increased IL-6 levels in H1N1pdm-infected mice, which was associated with disease pathogenesis. Interestingly, BALB/cJ mice were the weakest producers of IL-6. While we observed no statistical differences in disease severity between mouse strains, Otte et al. reported milder disease in BALB/cJ mice. Taken together, these studies may suggest IL-6 to be an indicator of disease severity in mice, as we observed in human patients. IL-6 deficiency did not impact disease outcome in C57BL/6J mice, but the consistent association of IL-6 with severe disease suggests host response elements that contribute to disease may be regulated by a common upstream event which also governs the IL-6 associated inflammatory response. Thus, IL-6 may serve as a potential disease severity biomarker during H1N1pdm infection, as IL-6 has been shown to be a biomarker in other diseases [15], [42]. Further investigation is critical to elucidate the role of IL-6 as a disease severity marker in influenza, specifically severe H1N1pdm.

Certain medical conditions such as asthma influence the host immune response and are risk factors for complication in H1N1pdm infection [6]. IL-6 may contribute differently to the inflammatory response in individuals with such comorbidities. Our models did not permit investigation of the role of IL-6 in individuals with comorbidities since C57BL/6J, BALB/cJ, and B6129SF2/J genetic backgrounds are all healthy, immunocompetent mouse models. Testing the contribution of IL-6 to disease in mice with underlying comorbidities, such as diabetes, may reveal roles for IL-6 in H1N1pdm pathogenesis not seen in our healthy mouse model. It is also possible our findings are limited to our mouse model and not representative of the host response in humans, although both species showed significant IL-6 induction following infection.

Importantly, we have shown increased IL-6 expression in humans hospitalized with H1N1pdm infection, supported by prominent IL-6 responses in mouse models of severe H1N1pdm infection, heavily implicating IL-6 in the host response to H1N1pdm. Yet, IL-6 deficiency in mice had no significant bearing on any of the clinical manifestations studied, suggesting that IL-6 does not play an essential non-redundant role in H1N1pdm infection in mice. Clinically, our study suggests IL-6 may serve as an important biomarker for the identification of patients at risk of severe complications following H1N1pdm infection, but may not be a suitable therapeutic target.

## Materials and Methods

### Ethics Statement

For clinical studies, written informed consent was obtained directly from each study participant or their legal representative before enrolment. Permission to perform scientific studies was granted by the Comisiόn de Investigaciόn del Hospital Clinico Universitario de Valladolid, Spain. Scientific and ethical approval of the study protocol was also obtained from the Scientific Committees for Clinical Research of each one of the participant centres.

For animal studies, all work was performed in strict accordance with the Canadian Council of Animal Care guidelines. Animal use protocols were approved by the Animal Care Committee of the University Health Network (Toronto, Canada). The University Health Network is certified under the Animals for Research Act (Permit Numbers: #0045 and #0085 under the jurisdiction of the Ontario Ministry of Agriculture, Food, and Rural Affairs) and follows NIH guidelines (OLAW #A5408-01). All infections and sample collections were performed under 5% isofluorane anaesthesia with every effort made to minimize suffering.

### Clinical Sample Collection

Hospitalized patients were recruited during the 2009 pandemic in ten different hospitals within the National Public Health System of Spain. Only those patients with confirmed H1N1pdm infection by qRT-PCR were included in the study. Viral RNA from nasopharyngeal swabs was obtained using automatic extractors purchased from either Biomérieux® (Marcy l’Etoile, France) or Roche® (Basel, Switzerland). Viral presence was then assessed in the Microbiology Services of the participant hospitals by qRT-PCR using either the Influenza A (H1N1) MGB Detection Assay from Applied Biosystems (Carlsbad, CA, USA), provided free of charge by the Centers for Disease Control (Atlanta, GA, USA), or the RealTime Ready Influenza A/H1N1 Detection Set from Roche® (Basel, Switzerland). Patients with signs of bacterial infection defined by the presence of purulent respiratory secretions, and/or positive result in respiratory cultures, blood cultures, and/or positive urinary antigen test to *Legionella pneumophila* or *Streptococcus pneumoniae* were excluded from analysis.

A total of 45 hospitalized patients with laboratory-confirmed H1N1pdm were enrolled in the study. Patients were divided into two groups along two different criteria: the requirement of critical (n = 35) or non-critical (n = 10) care and survival (n = 35) or death (n = 10). A single serum sample was collected from each patient within 48 hours of hospital admission, according to a unified protocol for all the participant centers. Samples were adequately centrifuged and the serum was separated and stored at −80°C until immune mediator profiling.

### Serum IL-6 Measurement

Serum IL-6 levels were measured using the Biorad Luminex Assay Platform (Hercules, CA, USA) at the Infection & Immunity Medical Investigation Unit, HCUV-IECSCYL (Valladolid, Spain).

### Animals

Female C57BL/6J, BALB/cJ, B6129SF2/J, and B6.129S2-*Il6^tm1Kopf^*/J (IL-6−/−) mice (8–10 weeks of age) were purchased from Jackson Laboratories (Bar Harbor, MN, USA) and used in influenza studies. Notably, B6.129S2-*Il6^tm1Kopf^*/J (IL-6−/−) were generated from C57BL/6J blastocysts and bred to C57BL/6J mice for 11 generations. Mice were maintained on standard animal feed and water *ad libitum* in clean environmental conditions and controlled temperature and humidity with a 12 hour light and dark cycle. For infection studies, animals were housed in HEPA-filtered cage racks adherent to ABSL2+ conditions (Toronto General Hospital Animal Resource Centre, Toronto, Canada). All animal procedures were performed in a certified class II biosafety cabinet (Baker Company, Sanford, NC, USA).

### Viral Infection

All infection experiments were conducted with H1N1pdm strain, A/Mexico/4108/2009 (H1N1pdm), provided by the Centers for Disease Control and Prevention (Atlanta, GA, USA). Virus was propagated and titrated in embryonated eggs prior to animal challenge. Viral stocks were stored in liquid nitrogen and thawed prior to use. Mice (either C57BL/6J, BALB/cJ, B6129SF2/J, or IL-6−/−) were weighed and randomly assigned for sample collection. Mice were infected through intranasal instillation with 50 µL phosphate-buffered saline (mock infection) or 50 µL A/Mexico/4108/2009 (H1N1pdm) at 1×10^5^ or 1×10^4^ 50% egg infectious dose (EID)_50_. Virus dosage was 1×10^5^ EID_50_ for host response profiling in C57BL/6J mice and 1×10^4^ EID_50_ for comparing disease severity between C57BL/6J, BALB/cJ, and B6129SF2/J mice, as well as between C57BL/6J and IL-6−/− mice. Throughout infection experiments, animal survival, clinical signs, and weights were recorded daily. In accordance with Animal Care Committee recommendation, mice were euthanized when recorded body weight fell below 80% of original body weight.

### Viral Load Measurement

At days 0 and 3 pi, mice (n = 3) were euthanized and lung homogenates collected for viral load determination. Lungs were homogenized (10% w/v) in High Glucose (4.5 g/L) Dulbecco’s Modified Eagle Medium (DMEM), supplemented with 1% bovine serum albumin, 50 µg/mL Gentamycin, 100 U/mL Penicillin, 100 µg/mL Streptomycin, and 1 µg/mL TPCK-Trypsin (vDMEM). Homogenates were then serially diluted (0.5 log_10_) in quadruplicate over Madin-Darby Canine Kidney cells, cultured at 2.0×10^4^ cells/well in 96-well plates. Cells were incubated for 2 hours at 37°C and 5% CO_2_. Homogenates were then removed and replaced with fresh vDMEM. Cells thus infected were incubated for 6 days at 37°C and 5% CO_2_, after which cell culture supernatants were tested for the presence of virus by hemagglutination assay using 0.5% (v/v) turkey red blood cells (LAMPIRE Biological Laboratories, Pipersville, PA, USA). Viral loads were determined as the reciprocal of the dilution at which 50% of wells were positive for viral infection. Viral loads were reported as TCID_50_ per gram of lung tissue. Limit of detection of 10^1.75^ TCID_50_/g.

### Cytokine Protein Measurement

At days 0 and 5 pi, lung tissues and serum were collected for cytokine measurement (n = 3 for each). Lungs were homogenized (10% w/v) in High Glucose (4.5 g/L) DMEM supplemented with cOmplete EDTA-free protease inhibitor cocktail (Roche Applied Science, Laval, Canada). Cytokine levels, including IL-6, were measured by cytokine bead array Mouse Inflammation Kit (BD Biosciences, San Diego, CA, USA), as per manufacturer’s instructions.

### Host Gene Expression Measurement by qRT-PCR

Total lung tissue RNA was reverse transcribed using ImProm-II Reverse Transcription System (Promega, Madison, WI, USA). qRT-PCR was performed using the ABI-Prism 7900HT Sequence Detection Systems (Applied Biosystems, Foster City, CA, USA). Raw data was collected with Applied Biosystems Sequence Detection Systems Version 2.2 software. Each reaction well contained 0.25 µL of 20 ng/uL cDNA, 0.5 µL each of forward and reverse primers (final concentration of 200 nM), and 5 µL SYBR Green Master Mix, for a total reaction volume of 10 uL. Each sample was run in quadruplicate. Gene expression was normalized to the β-actin housekeeping gene, and quantified by the relative standard curve method. Primer sequences are listed in Table S2.

### Microarray Analysis

At designated time points (ie. days 0 through 6 pi), C57BL/6J mice were euthanized (n = 3) and lung tissue was collected. Total RNA was amplified with Illumina TotalPrep RNA Amplification Kit (Ambion, Austin, TX, USA) as per manufacturer’s instructions. cRNA (1.5 µg) was labelled and hybridized to MouseWG-6 v2.0 Expression BeadChip (Illumina, San Diego, CA, USA) and scanned on Illumina BeadStation 500GX. Raw data was collected with Illumina GenomeStudio V2010.3 software. The data sets were pre-processed with quantile normalization, variance stabilization, and log_2_ transformation. Time series analysis was performed with Extraction of Differential Gene Expression (EDGE) software (Storey Lab, University of Washington, WA, USA). Benjamini-Hochberg correction was employed to assess the occurrence of false positives by calculating the false discovery ratio. Hierarchical clustering by Pearson’s correlation and heatmap representations were generated using MultiExperiment Viewer v4.6.2 (Dana-Farber Cancer Institute, Boston, MA, USA). Ingenuity Pathway Analysis v9.0 (IPA) (Ingenuity Systems, Redwood City, CA, USA) was used to select, annotate and visualize genes by function and pathway. DAVID Bioinformatics Resource v6.7 [27], [28] was also used to perform functional classification of differentially expressed genes.

### Histology

C57BL/6J mice and IL-6−/− mice were euthanized at day 3 (n = 3) and day 6 (n = 1) pi and harvested lung tissues were perfused with 10% buffered formalin. Tissues were paraffin wax embedded for histology. Sections were stained with Hematoxylin and Eosin and observed under light microscope (Accu-scope®, Commack, NY, USA). Images were captured using a digital camera and SE Premium software (Micrometrics™, Londonderry, NH, USA).

### Microarray Data Accession Number

Microarray expression data are MIAME compliant and are available publicly in the MIAME-compliant Gene Expression Omnibus database (www.ncbi.nlm.nih.gov/geo/) under accession number GSE31022.

### Statistical Methods

For clinical patient data, serum IL-6 levels were compared using the Mann-Whitney U test (α = 0.05). BLR analysis was employed to predict the probability of critical care admission and death as a function of IL-6 (log_10_ IL-6 (pg/mL)) levels, adjusted by age and sex. Both age and sex were identified as confounding variables when investigating the relationship between IL-6 levels and disease severity. Adjustment was performed by incorporating age and sex as predictors in the logistic regression model, thereby minimizing the confounding effect of these variables. The Student’s t-test (α = 0.05) was used to identify statistically significant increases in expression of genes assayed by qRT-PCR. Survival curves were compared by the logrank test (α = 0.05) [43]. Comparisons of weight loss, viral load, gene fold change in expression, and cytokine expression were also made using the Student’s t-test (α = 0.05). All Student’s t-tests assumed two-tailed distributions and two-sample unequal variances.

## Supporting Information

Figure S1 **Mortality and weight loss in C57BL/6J mice infected A/Mexico/4108/2009 (H1N1pdm).** Survival curve for C57BL/6J mice infected intranasally with 10^5^ EID_50_ A/Mexico/4108/2009 (H1N1pdm). n = 35 (A). Average weight curve for C57BL/6J mice infected intranasally with 10^5^ EID_50_ A/Mexico/4108/2009 (H1N1pdm). Vertical error bars indicate ±1 standard deviation for the average weight at each time point. n = 35 (B).
(TIF)

Table S1 **Increased expression of acute inflammatory response genes in C57BL/6J mice infected with A/Mexico/4108/2009 (H1N1pdm).** DAVID Bioinformatics Resource v6.7 [27], [28] was applied to all differentially expressed genes for classification. Gene Ontology Biological Process pathway GO:0002526 – acute inflammatory response was selected as a suitable representation of genes implicated in the acute phase response. Fold changes in expression are expressed in log_2_. day 0 values normalized to 0.
(DOC)

Table S2 **Sequences of primers used for qRT-PCR.**
(DOC)
