# Supplementary figures and images for: Interleukin-6 Is a Potential Biomarker for Severe Pandemic H1N1 Influenza A Infection
Source: PLoS One. 2012 Jun 5;7(6):e38214. doi: 10.1371/journal.pone.0038214 (PMC3367995; doi:10.1371/journal.pone.0038214)

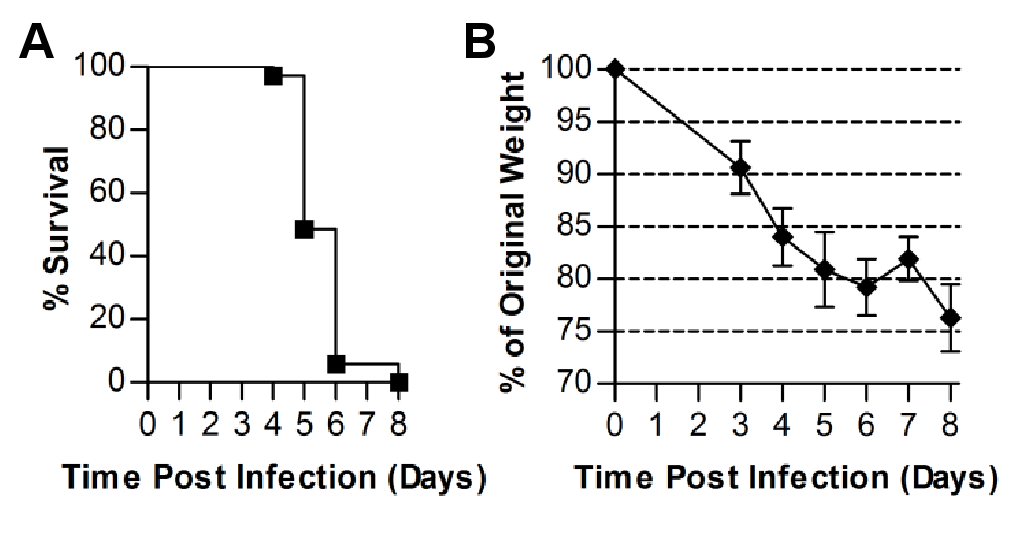

Supplement: Figure S1 — Mortality and weight loss in C57BL/6J mice infected A/Mexico/4108/2009 (H1N1pdm). Survival curve for C57BL/6J mice infected intranasally with 105 EID50 A/Mexico/4108/2009 (H1N1pdm). n = 35 (A). Average weight curve for C57BL/6J mice infected intranasally with 105 EID50 A/Mexico/4108/2009 (H1N1pdm). Vertical error bars indicate ±1 standard deviation for the average weight at each time point. n = 35 (B). (TIF) [file pone.0038214.s001.tif]
