# Supplementary material for: Interleukin-6 Is a Potential Biomarker for Severe Pandemic H1N1 Influenza A Infection
Source: PLoS One. 2012 Jun 5;7(6):e38214. doi: 10.1371/journal.pone.0038214 (PMC3367995; doi:10.1371/journal.pone.0038214)
Supplement: Table S1 — Increased expression of acute inflammatory response genes in C57BL/6J mice infected with A/Mexico/4108/2009 (H1N1pdm). DAVID Bioinformatics Resource v6.7 [27], [28] was applied to all differentially expressed genes for classification. Gene Ontology Biological Process pathway GO:0002526 – acute inflammatory response was selected as a suitable representation of genes implicated in the acute phase response. Fold changes in expression are expressed in log2. day 0 values normalized to 0. (DOC) [file pone.0038214.s002.doc]

**Table S1. Increased expression of acute inflammatory genes in C57BL/6J mice infected with** A/Mexico/4108/2009 (H1N1pdm).

| **Pathway and Official Gene Symbol** | **Name or Description** | **Illumina Identification** | **Day 1** | **Day 3** | **Day 6** |
| --- | --- | --- | --- | --- | --- |
| **GO:0002526 – acute inflammatory response** | | |  |  |  |
| Saa3 | serum amyloid A 3 | ILMN_2772632 | **3.08** | **6.73** | **6.63** |
| CFB | complement factor B | ILMN_1230853 | **1.32** | **3.23** | **3.54** |
| fcgr1 | Fc receptor, IgG, high affinity I | ILMN_1229523 | **0.71** | **3.21** | **3.84** |
| Serpina3n | serine (or cysteine) peptidase inhibitor, clade A, member 3N | ILMN_1246800 | **0.86** | **3.07** | **2.39** |
| IDO1 | indoleamine 2,3-dioxygenase 1 | ILMN_1223600 | 0.15 | **2.73** | **5.10** |
| IL1B | interleukin 1 beta | ILMN_2777498 | **0.72** | **2.45** | **2.93** |
| ORM2 | orosomucoid 2 | ILMN_2591264 | 0.04 | **2.44** | **1.51** |
| IL6 | interleukin 6 | ILMN_1243601 | 0.29 | **2.16** | **2.37** |
| LOC100045296, Stat3 | similar to Stat3B; signal transducer and activator of transcription 3 | ILMN_2589790 | **1.45** | **1.86** | **1.05** |
| C2 | complement component 2 (within H-2S) | ILMN_2612895 | **0.82** | **1.71** | **1.52** |
| TLR4 | toll-like receptor 4 | ILMN_2684234 | **0.90** | **1.39** | **1.49** |
| SAA4 | serum amyloid A 4 | ILMN_1247506 | -0.22 | **1.29** | **1.35** |
| Il1a | interleukin 1 alpha | ILMN_1243066 | -0.04 | **0.99** | **1.15** |
| FCGR3 | Fc receptor, IgG, low affinity III | ILMN_2687403 | 0.40 | **0.87** | **1.52** |
| TLR4 | toll-like receptor 4 | ILMN_2752966 | 0.51 | **0.79** | **1.09** |
| ADORA3 | adenosine A3 receptor | ILMN_3061260 | -0.05 | **0.79** | **1.32** |
| LOC100045680, C4a, LOC675521, C4b | similar to Complement C4 precursor; complement component 4A (Rodgers blood group); similar to complement C4; complement component 4B (Childo blood group) | ILMN_3049559 | 0.55 | **0.78** | **1.17** |
| CFH, LOC100048018 | complement component factor h; similar to complement component factor H | ILMN_2592066 | 0.42 | **0.65** | **0.80** |
| C3, LOC100048759 | complement component 3; similar to complement component C3 prepropeptide, last | ILMN_2759484 | 0.34 | 0.42 | **0.61** |
| Igh-3, Ighg | immunoglobulin heavy chain 3 (serum IgG2b); Immunoglobulin heavy chain (gamma polypeptide) | ILMN_1222531 | -0.28 | -0.28 | -0.36 |
| Hc | hemolytic complement | ILMN_2659143 | **0.90** | -0.35 | -0.11 |
| Fcnb | ficolin B | ILMN_2663083 | -0.41 | -0.38 | -0.20 |
| LOC100045680, C4a, LOC675521, C4b | similar to Complement C4 precursor; complement component 4A (Rodgers blood group); similar to complement C4; complement component 4B (Childo blood group) | ILMN_2632563 | -0.67 | -0.57 | -0.53 |
| SIGIRR | single immunoglobulin and toll-interleukin 1 receptor (TIR) domain | ILMN_2673121 | -1.45 | -1.58 | -2.14 |

DAVID Bioinformatics Resource v6.7 was applied to all differentially expressed genes for classification. Gene Ontology Biological Process pathway GO:0002526 – acute inflammatory response was selected as a suitable representation of genes implicated in the acute phase response. Fold changes in expression are expressed in log_2_. day 0 values normalized to 0.
