# Supplementary material for: Interleukin-6 Is a Potential Biomarker for Severe Pandemic H1N1 Influenza A Infection
Source: PLoS One. 2012 Jun 5;7(6):e38214. doi: 10.1371/journal.pone.0038214 (PMC3367995; doi:10.1371/journal.pone.0038214)
Supplement: Table S2 — Sequences of primers used for qRT-PCR. (DOC) [file pone.0038214.s003.doc]

**Table S2. Sequences of primers used for qRT-PCR**

| **Target Gene** | **NCBI Accession Number** | **Primer Sequences** |
| --- | --- | --- |
|  |  |  |
| *Il6* | NM_031168.1 | Forward: 5’-CTTCAGAGAGATACAGAAACTCTAAT-3’  Reverse: 5’-GCTTATCTGTTAGGAGAGCAT-3’ |
| *Stat3* | NM_213659.2 | Forward:5’-ATGAAGGTGGTGGAGAAC-3’  Reverse: 5’-CTGCATCTTCTGTCTGGTC-3’ |
| *Il6ra* | NM_010559.2 | Forward: 5’-TGGATAGCAGAGCCCAGGACCA-3’  Reverse: 5’-GGGCGAGGACACTCGTTGCT-3’ |
| *Orm2* | NM_011016.2 | Forward: 5’-GACCCTGAGCTGGCTCTCTGAC-3’  Reverse: 5’-TCAGGGTTTAGGACAGCCGCAC -3’ |
| *Saa3* | NM_011315.3 | Forward: 5’-CAGCACGAGCAGGATGAAGCCTT-3’  Reverse: 5’-TGTCAGAGTAGGCTCGCCACATGT-3’ |
| *Saa4* | NM_011316.3 | Forward: 5’-TGAGGCTTGCCACCGTCATTGT-3’  Reverse: 5’-AGTCCCAAGTCCCTTGTACGGCT-3’ |
| *Socs1* | NM_009896.2 | Forward: 5’-CAGCGGCACGGCTCCCA-3’  Reverse: 5’-TAGCGTCCGGGGCTGCG-3’ |
| *Socs3* | NM_007707.3 | Forward: 5’-GAGATTTCGCTTCGGGACTA-3’  Reverse: 5’-GGAAACTTGCTGTGGGTGA-3’ |
| β-Actin (*Actb*) | NM_007393.3 | Forward: 5’-AGGGAAATTGTTCGTGACATAAA-3’  Reverse: 5’-TCATAACTCTTCTCCAAGGAGG-3’ |
|  |  |  |
